# The genomics of domestication special issue editorial

**DOI:** 10.1111/eva.12693

**Published:** 2018-09-17

**Authors:** Michael B. Kantar, Michael W. Bruford, Loren H. Rieseberg

**Affiliations:** ^1^ Department of Tropical Plant and Soil Sciences University of Hawai'i Honolulu Hawaii; ^2^ School of Biosciences Cardiff University Cardiff UK; ^3^ Sustainable Places Research Institute Cardiff University Cardiff UK; ^4^ Department of Botany and Biodiversity Research Centre University of British Columbia Vancouver British Columbia Canada

## Abstract

Domestication has been of major interest to biologists for centuries, whether for creating new plants and animal types or more formally exploring the principles of evolution. Such studies have long used combinations of phenotypic and genetic evidence. Recently, the advent of a large number of genomes and genomic tools across a wide array of domesticated plant and animal species has reinvigorated the study of domestication. These genomic data, which can be easily generated for nearly any species, often provide great insight with or without a reference genome. The comparison of genome wide data from domestic and wild species has ignited a wave of insight into human, plant, and animal history with a new range of questions becoming accessible. With this in mind, this issue of *Evolutionary Applications* includes eleven papers covering a wide range of perspectives and methodologies relevant to understanding genomic variation under domestication.

This issue has five broad topics, with the first focused on deleterious mutations in the context of domestication (Bosse, Megens, Derks, de Cara, & Groenen, 2018; Plekhanova, Nuzhdin, Utkin, & Samsonova, 2018). Bosse et al. (2018) use data from pig and chickens to explore mutational load, finding that domestication does not necessitate a reduction in fitness, but rather specific species‐level factors impact the extent to which deleterious mutations accumulate in domestic populations. They make the important point that whether a mutation is deleterious or not may be context dependent. That is, alleles that are deleterious in wild populations may be neutral or even slightly beneficial in a domestication setting or in another species. In the second paper on this topic, Plekhanova and colleagues develop a new comparative genomics tool that to distinguish among mutations that are likely to be generally deleterious and those whose fitness effects are predicted to be context dependent. Their approach capitalizes on human genetic disease data and transfer learning to classify such mutations in common pet/farm animals (Plekhanova et al., 2018). Classifying the extent of genetic load in domestic plants and animals provides new ways for understanding why certain populations collapse, how fitness changes in different types of populations, and has profound implications for breeding efficiency in domestic populations.

The second topic covered is the genomics of ancient samples and its important contribution to understanding domestication (Allaby, Ware, & Kistler, 2018; Wales et al., 2018). In the first article in this section, Wales et al. (2018) explore archeological plastids in sunflower comparing these ancient samples with historic landraces and modern cultivars, identifying a relatively small bottleneck during domestication and large bottleneck during modern breeding (Wales et al., 2018). These data show how quickly it is possible to alter diversity within a species, and how much genetic loss in domestic species can be accounted for by recent commercial breeding, a phenomenon common to animals and plants (Taberlet et al., 2008). Further, Allaby et al. (2018) use a time series of archeological sequence data from three species (barley, maize, and sorghum) to explore the genetic bottleneck associated with domestication. In each of these species, bottlenecks appear to be quite weak early in the domestication process, suggesting that even when single domestication events occurred, populations were not as small as originally thought. These data combine to give an unprecedented time series of material to see how domestication occurred in real time and are providing new insights, suggesting, for example, that the “domestication bottleneck paradigm may not be as universal as once thought.” This has been shown most recently in goats, where a “mosaic” of events appears to be the most likely scenario in goat domestication (Daly et al., 2018).

The third section concerns introgression between wild and domestic populations (Owens, Baute, Hubner, & Rieseberg, 2018; Schreiber, Himmelbach, Börner, & Mascher, 2018; Taitano et al., 2018; Vigueira et al., 2017). The first of these studies authored by Vigueira et al. (2017) explores weedy rice inside and outside the rice native range, to understand diverse mechanisms for de‐domestication. Not surprisingly, introgression from wild to weedy rice was found to play an important role in weed evolution in geographic regions where the two taxa co‐occur, but not where the wild progenitor is absent. This implies that management decisions as well as genetic mechanisms of de‐domestication spatially vary. In sunflower, Owens et al. (2018) analyze a late phase in domestication, hybrid crop development, which involved widespread introgression from wild relatives as populations were partitioned into male and female lines. Further, they showed that there was minimal selection for overdominance, but that selection was centered around branching and fertility/sterility mechanisms. In a new collection of wild and landrace chili pepper from Mexico, Taitano et al. (2018) explore how historic uses appeared to partition the population within the native range, but that this use type partitioning broke down when comparisons were extended to a worldwide pepper collection where there was admixture between different use types in the native range. Lastly, Schreiber et al. (2018) examine the secondary domesticated species rye, while not part of the original Neolithic package it nevertheless has become an important staple in much of Eurasia. They find that there is little genetic differentiation between domestic and wild rye, likely indicating large‐scale gene flow within the native range. Together, these papers explore the ongoing processes of local adaptation and how domestication has been shaped by proximal wild populations, particularly within the species native range.

The fourth section explores demographic processes in domestication (Pitt, Bruford et al., 2018; Pitt, Sevane et al., 2018;). Pitt, Sevane et al. (2018) examine prevailing hypotheses on different numbers of domestication events in cattle using approximate Bayesian computation, identifying two domestication events as the most likely scenario with admixture having likely occurred between African and Asian cattle, explaining their shared genetic variation. In another contribution, Pitt, Bruford et al. (2018) explore how creole cattle underwent serial demographic fluctuations in Colombia, based on changing human trait preference and changes in local breed preferences, again highlighting how large changes in effective population size can occur over short time frames. Further, they identified genes that have led to adaptation to tropical humid environments (linking to the last topic in this special issue), different from their ancestors’ Iberian conditions. Both of these papers explore the basic question of how populations diverge over time and take on different characteristics relative to the traits under human selection.

The last topic in this special issue is the use of genomics in detecting selection in domesticated species, in which López et al. (2018) use complementary statistical approaches to detect selection in salmon. These methods had limited overlap, suggesting that a single method is insufficient to describe populations and that drift appears to be more important than selection in salmon domestication. However, this result may be an artifact of domestication targeting polygenic traits in fish. Understanding the best way to detect selection is still central to our desire to understand how humans have impacted their favorite species.

Understanding genomewide dynamics provides new insights into all of these and many more topics, by providing a finer‐grained understanding of how the genome changes and how these changes persist through known states of population differentiation. Comparative domestication studies of plants and animals using multiple techniques for understanding genomic change, as well as studying different types of populations (historic and extant), allow us to learn ever more about the human condition. Together, these articles demonstrate the emergence of a new understanding of how genomewide effects may differ from those at specific loci and how these effects can change dramatically over relatively fast time scales. It also shows how studies of the process of domestication provide tremendous insight into the change in fitness in populations and can be used to improve plant and animal breeding populations and inform other areas of evolutionary biology such as conservation and adaptation in the wild.

## CONFLICT OF INTEREST

None declared.
